# Neoadjuvant camrelizumab plus chemotherapy in treating locally advanced esophageal squamous cell carcinoma patients: a pilot study

**DOI:** 10.1186/s12957-021-02446-5

**Published:** 2021-11-22

**Authors:** Peng Yang, Xiao Zhou, Xuefeng Yang, Yuefeng Wang, Tao Sun, Shiying Feng, Xianyou Ma

**Affiliations:** 1 Department of Cardiothoracic Surgery, Daqing Oilfield General Hospital, Daqing, 163000 Heilongjiang China; 2 Department of Oncology, Daqing Oilfield General Hospital, No. 9 Zhongkang Street, Saertu District, Daqing, 163000 Heilongjiang China

**Keywords:** Camrelizumab, Chemotherapy, Neoadjuvant therapy, Esophageal squamous cell carcinoma, Efficacy and safety

## Abstract

**Background:**

Camrelizumab (a PD-1 inhibitor) has been used as a potential therapy in unresectable advanced esophageal squamous cell carcinoma (ESCC) along with adjuvant treatment in locally advanced ESCC, exhibiting an acceptable efficacy and safety profile. This pilot study was designed to further investigate the clinical value and tolerance of neoadjuvant camrelizumab plus chemotherapy in locally advanced ESCC.

**Methods:**

A total of 16 patients with locally advanced ESCC were recruited. Patients received 2 cycles of neoadjuvant therapy including 2 doses of camrelizumab concurrent with 2 cycles of paclitaxel plus carboplatin followed by surgery 4 weeks afterward. Then, the treatment response after neoadjuvant therapy, R0 resection rate, tumor regression grade (TRG), and pathological complete remission (pCR) rate were measured. Besides, adverse events were documented. At last, progression-free survival (PFS) and overall survival (OS) were assessed.

**Results:**

Generally, objective remission rate (ORR) was 81.3% whereas disease control rate (DCR) was 100% after neoadjuvant therapy. Concerning TRG grade, 31.3, 37.5, 18.8, and 12.5% patients reached TRG0, TRG1, TRG2, and TRG3, respectively. Then, pCR rate and R0 resection rate were 31.3 and 93.8%, respectively. Besides, mean PFS and OS were 18.3 months (95%CI: (16.2–20.5) months) and 19.2 months (95%CI: (17.7–20.7) months), respectively, with a 1-year PFS of 83% and OS of 90.9%. Adverse events included white blood cell decrease (37.5%), neutrophil decrease (31.3%), reactive cutaneous capillary endothelial proliferation (37.5%), and nausea or vomiting (25.0%), which were relatively mild and manageable.

**Conclusion:**

Neoadjuvant camrelizumab plus chemotherapy exhibits good efficacy and acceptable tolerance in patients with locally advanced ESCC.

## Introduction

Esophageal squamous cell carcinoma (ESCC), with a proportion of 87% in all esophageal cancer, exhibits a more extensive intratumor heterogeneity and a higher mortality [1–3]. Surgery is the most common treatment modality for the ESCC patients; however, as to patients with locally advanced ESCC, they always lose surgical opportunities because of the large tumor size. Fortunately, the emergence of neoadjuvant therapy not only decreases the tumor size but also brings more surgical opportunities to the patients with locally advanced ESCC [4, 5].

Chemotherapy is the first prior neoadjuvant therapy recommended by several guidelines [6–8] for these patients. Recently, several researches indicate that some certain targeted drugs (such as apatinib and nivolumab) in combination with chemotherapy probably promote the survival profile in locally advanced ESCC [9–11].

Programmed cell death protein 1 (PD-1), expressed by activated lymphocytes, blocks immune responses and facilitates immune escape by binding to its ligands including programmed cell death ligand 1 (PD-L1), which further contributes to tumorigenesis and disease progression in various malignancies [12, 13]. PD-1 inhibitor, as a novel developed immune therapy, blocks the PD-1/PD-L1 linkage and has been widely applied in numerous carcinomas [12, 14]. In addition, in locally advanced ESCC, nivolumab and pembrolizumab combination with chemotherapy was recently used for neoadjuvant therapy, which exhibited an acceptable therapeutic response, progression-free survival (PFS), and overall survival (OS) [9]. Camrelizumab, a domestic product developed in China, is a novel IgG4-kappa anti-PD-1 inhibitor that has been used for treatment of a variety of malignancies, such as refractory classical Hodgkin’s lymphoma and gastric or gastroesophageal junction adenocarcinoma [15, 16]. Moreover, camrelizumab has been previously witnessed for adjuvant or second-line therapy of locally advanced ESCC [17, 18]. However, the clinical value of neoadjuvant camrelizumab plus chemotherapy in the treatment for locally advanced ESCC has not been reported before.

Thus, this study aimed to explore the therapeutic response, survival, and safety profiles of neoadjuvant camrelizumab plus chemotherapy in treating locally advanced ESCC.

## Methods

### Patients

The current study was a prospective study. A total of 16 patients with locally advanced ESCC who were recruited between July 1, 2019, and December 31, 2020, and received camrelizumab combined with chemotherapy as neoadjuvant therapy before surgery in our hospital. The inclusion criteria were as follows: (i) histologically confirmed as ESCC; (ii) potentially curable and locally advanced ESCC, which was defined as cT1N1-3M0 or cT2-4aN0-3M0 (Union for International Cancer Control, UICC Version 8.0) [19]; (iii) aged >18 years old; (iv) Eastern Cooperative Oncology Group performance status (ECOG PS) of 0 to 1; (v) willing to receive the neoadjuvant therapy of camrelizumab combined with paclitaxel and carboplatin; and (vi) able to be regularly follow-up. The exclusion criteria were as follows: (i) poorly controlled underlying diseases or insufficient hepatic, hematological, and kidney functions that led to be unsuitable for neoadjuvant therapy; (ii) hypersensitivity to the study drugs; (iii) difficult to perform gastric tube reconstruction after esophagectomy; (iv) known concurrent malignancies; (v) severe infections; (vi) history of use of camrelizumab, paclitaxel or carboplatin; and (vii) pregnancy. Ethical permission for this study was obtained from the Institutional Review Board with the approval number of KS1951 on 11th May 2019, and the written informed consents were acquired from all patients. Our study was registered on Chinese Clinical Trial Registry (https://www.chictr.org.cn/index.aspx) with the approval number of “ChiCTR2100051903”.

### Neoadjuvant therapy and surgery

All patients received 2 cycles of neoadjuvant therapy, including 2 doses of camrelizumab concurrent with 2 cycles of paclitaxel plus carboplatin. In detail, the camrelizumab was administered intravenously at a dose of 200 mg each time, every 3 weeks (a treatment cycle). Simultaneously, the paclitaxel was administered by intravenous drip at a dose of 100 mg/m^2^ of body-surface area on days 1 and 8, and the carboplatin was administered by intravenous drip and targeted at an area under the curve of 5 mg/ml per minute on day 1. The surgery was performed approximately 4 weeks after completion of 2 cycles of neoadjuvant therapy, and the type of surgery included minimally invasive esophagectomy, right transthoracic open esophagectomy, or hybrid approaches (using video-assisted thoracoscopy and laparotomy) with a total 2-field lymphadenectomy. Complete thoracoabdominal two-field lymph node dissection (standard thoracoabdominal two-field plus mediastinum, especially bilateral recurrent laryngeal nerve chain lymph nodes) was performed for the patients without suspicious enlarged lymph nodes in the neck and the patients with middle and lower thoracic esophageal cancer. Cervical-thoracoabdominal three-field lymph node dissection (upper and lower neck and supraclavicular complete two-field lymph nodes mentioned above) was performed for the patients with suspiciously swollen lymph nodes in the neck and patients with upper thoracic esophageal cancer. As for adjuvant therapy, patients received paclitaxel plus carboplatin regimen for about 2–4 cycles.

### Treatment response evaluation and safety assessment

The primary endpoint was pathological complete remission (pCR), and the secondary endpoints included treatment response, tumor regression grade (TRG), R0 resection rate, adverse events, progression-free survival (PFS), and overall survival (OS). After the completion of 2 cycles of neoadjuvant therapy, a preoperative computed tomography (CT) examination was performed to assess the treatment response according to the Response Evaluation Criteria In Solid Tumors (RECIST Version 1.1) [20]. Meanwhile, objective remission rate (ORR) and disease control rate (DCR) were calculated as follows: ORR = complete remission (CR) rate plus partial remission (PR) rate and DCR = CR rate plus PR rate plus stable disease (SD) rate. After surgery, pathological examination of the resection specimens was conducted to evaluate the resection margin status and tumor regression grade (TRG). R0 resection was defined as no cancer cells at resection margins. The TRG was used for assessment of the degree of histomorphological degeneration, which was classified as follows: grade 0, no residual cancer cells (defined as pCR); grade 1, single cell or small groups of cancer cells; grade 2, residual cancer cells outgrown by fibrosis; and grade 3, minimum or no treatment effect and extensive residual cancer cells [21, 22]. In addition, adverse events occurred during the neoadjuvant therapy were documented in detail to access safety profiles, which consisted mainly of hematologic adverse events and non-hematologic adverse events.

### Survival assessment

For assessment of progression-free survival (PFS) and overall survival (OS), the postoperative surveillance and follow-up for patients were implemented every 3 months in the first year then every 6 months in the following years.

### Statistical analysis

Data processing and graph plotting were carried out with the use of SPSS 26.0 (IBM Corp., Armonk, NY, USA) and GraphPad Prism 6.02 (GraphPad Software Inc., San Diego, CA, USA). Descriptive analysis was completed with the use of the statistics including mean with standard deviation (SD), median with interquartile range (IQR), and frequency with percentage. Survival curves were plotted using Kaplan-Meier method.

## Results

### Baseline characteristics

In total, 16 patients with locally advanced ESCC were enrolled in present study (Table 1). The age was 60.9±7.8 years. Regarding gender, the number of male and female patients was 14 (87.5%) and 2 (12.5%), respectively. Moreover, 13 (81.3%) patients were scored as ECOG PS score 0, while 3 (18.8%) patients were scored as ECOG PS score 1. Besides, the distribution of tumor location was listed as follows: 3 (18.8%) patients with proximal third, 8 (50.0%) patients with middle third, and 5 (31.3%) patients with distal third. Furthermore, there were 0 (0.0%), 4 (25.0%), 10 (62.5%), and 2 (12.5%) patients were diagnosed as TNM stage I, stage II, stage III, and stage IV, respectively. The detailed characteristics were shown in Table 1.

**Table 1:** Characteristics of locally advanced ESCC patients

Characteristics	ESCC patients (*N* = 16)
Age (years)	
Median (IQR)	60.5 (56.0-67.3)
Mean±SD	60.9±7.8
Male, No. (%)	14 (87.5)
ECOG PS, no. (%)	
0	13 (81.3)
1	3 (18.8)
Tumor location, no. (%)	
Proximal third	3 (18.8)
Middle third	8 (50.0)
Distal third	5 (31.3)
Histological grade, no. (%)	
Well	1 (6.3)
Moderate	8 (50.0)
Poor	7 (43.7)
Clinical T stage, no. (%)	
cT1	0 (0.0)
cT2	2 (12.5)
cT3	13 (81.3)
cT4	1 (6.3)
Clinical N stage, no. (%)	
N0	2 (12.5)
N1	10 (62.5)
N2	3 (18.8)
N3	1 (6.3)
Clinical TNM stage, no. (%)	
Stage I	0 (0.0)
Stage II	4 (25.0)
Stage III	10 (62.5)
Stage IV	2 (12.5)

*ESCC* esophageal squamous cell carcinoma, *IQR* interquartile range, *SD* standard deviation, *ECOG PS* Eastern Cooperative Oncology Group performance status

### Treatment response

**Fig. 1:**
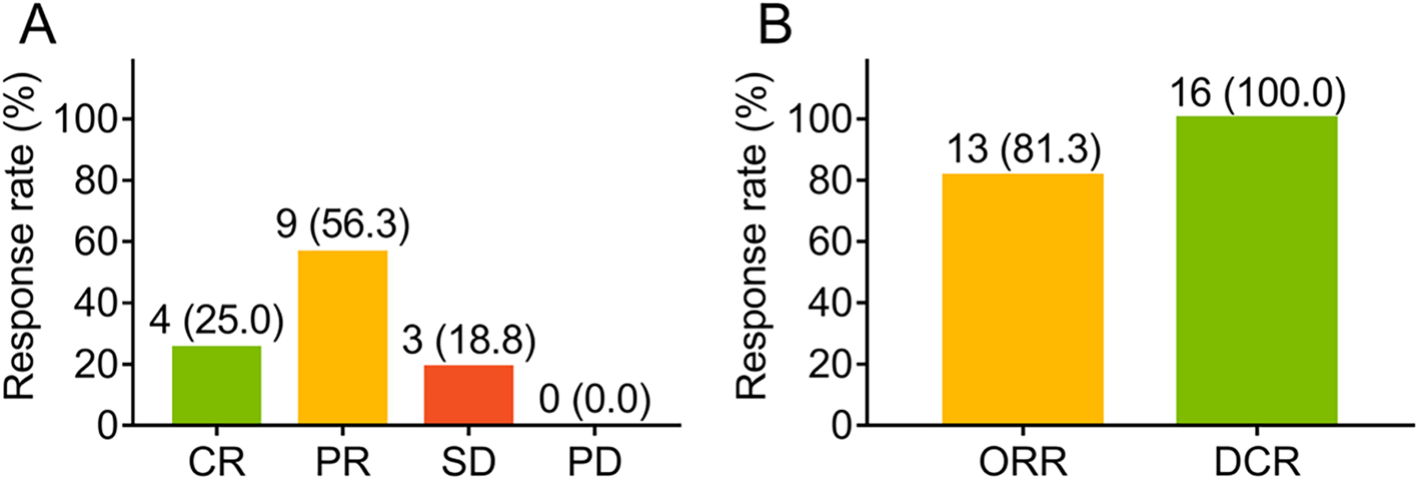
Treatment response rate in patients with locally advanced ESCC. Treatment response rate concerning CR, PR, SD, and PD (**a**). Treatment response rate concerning ORR and DCR (**b**) adopting neoadjuvant camrelizumab plus chemotherapy in locally advanced ESCC. CR, complete remission; PR, partly remission; SD, stable disease; PD, progressive disease; ORR, objective remission rate; DCR, disease control rate; ESCC, esophageal squamous cell carcinoma

**Fig. 2:**
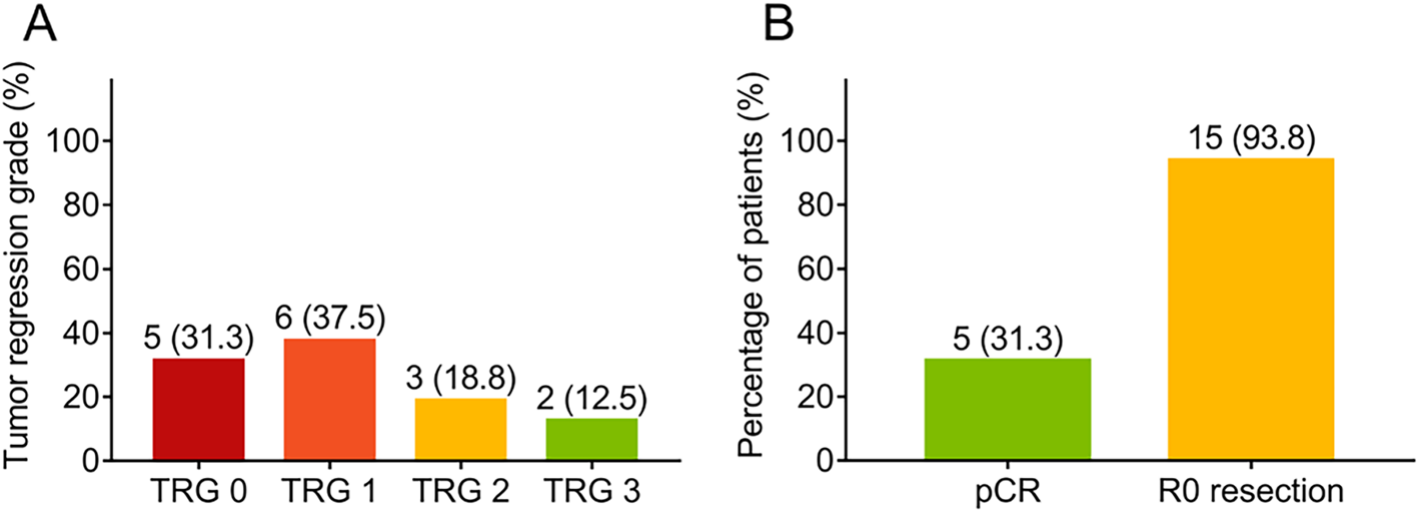
Pathological response rate in patients with locally advanced ESCC. TRG rate (**a**), pCR rate and R0 resection rate (**b**) of neoadjuvant camrelizumab plus chemotherapy in locally advanced ESCC. TRG, tumor regression rate; pCR, pathological complete response; ESCC, esophageal squamous cell carcinoma

After neoadjuvant therapy, 4 (25.0%) patients achieved CR, 9 (56.3%) patients reached PR and 3 (18.8%) patients remained SD, whereas no one got progressive disease (PD) (Fig. 1a). To sum up, ORR was 81.3%; meanwhile, DCR was 100% (Fig. 1b). Regarding tumor regression grade, 5 (31.3%), 6 (37.5%), 3 (18.8%), and 2 (12.5%) patients reached TRG 0, TRG 1, TRG 2, and TRG 3, respectively (Fig. 2a). Furthermore, 5 (31.3%) patients achieved pCR (Fig. 2b). Besides, 15 (93.8%) patients realized R0 resection.

### Survival profiles

**Fig. 3:**
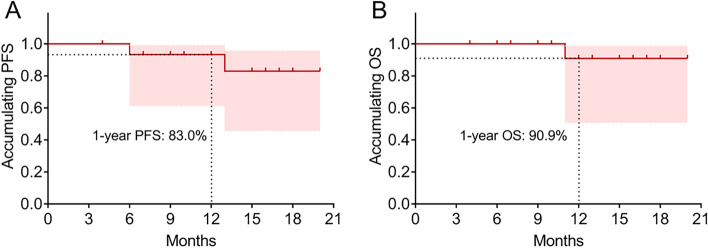
PFS and OS in patients with locally advanced ESCC. The PFS (**a**) and OS (**b**) adopting neoadjuvant camrelizumab plus chemotherapy in locally advanced ESCC. PFS, progression-free survival; OS, overall survival; ESCC, esophageal squamous cell carcinoma

The mean PFS was 18.3 months (95%CI: (16.2–20.5) months) with a 1-year PFS rate of 83% (Fig. 3a); meanwhile, the mean OS was 19.2 months (95%CI: (17.7–20.7) months) with a 1-year OS rate of 90.9% (Fig. 3b). In addition, the detailed information of each patient in terms to baseline characteristics, responses, and survival data was listed in Table 2.

**Table 2:** Key features and treatment outcomes of each advanced ESCC patient

No.	Age (years)	Gender	ECOG PS	Location	Clinical T stage	Clinical N stage	Clinical TNM stage	Treatment response	TRG	pCR	R0 resection	Progression	PFS (months)	Death	OS (months)
1	57	M	0	Proximal third	T3	N0	Stage II	CR	1	No	Yes	No	16	No	16
2	64	M	0	Proximal third	T2	N1	Stage II	CR	0	Yes	Yes	No	13	No	13
3	61	M	0	Middle third	T3	N1	Stage III	PR	0	Yes	Yes	No	20	No	20
4	65	M	0	Proximal third	T3	N2	Stage III	PR	2	No	Yes	No	18	No	18
5	52	M	0	Distal third	T2	N1	Stage II	SD	1	No	Yes	No	16	No	16
6	68	M	0	Middle third	T3	N1	Stage III	PR	0	Yes	Yes	No	7	No	7
7	70	M	0	Distal third	T3	N0	Stage II	PR	0	Yes	Yes	No	10	No	10
8	58	M	0	Middle third	T3	N1	Stage III	PR	1	No	Yes	No	17	No	17
9	53	M	1	Distal third	T3	N2	Stage III	PR	1	No	Yes	Yes	13	No	16
10	72	F	1	Middle third	T3	N3	Stage IV	SD	3	No	No	Yes	6	Yes	11
11	56	M	1	Middle third	T3	N1	Stage III	CR	1	No	Yes	No	9	No	9
12	46	M	0	Distal third	T3	N1	Stage III	PR	2	No	Yes	No	6	No	6
13	62	M	0	Distal third	T3	N2	Stage III	PR	1	No	Yes	No	12	No	12
14	60	F	0	Middle third	T3	N1	Stage III	CR	0	Yes	Yes	No	15	No	15
15	56	M	0	Middle third	T4	N1	Stage IV	PR	3	No	Yes	No	4	No	4
16	75	M	0	Middle third	T3	N1	Stage III	SD	2	No	Yes	No	16	No	16

*ESCC* esophageal squamous cell carcinoma, *ECOG PS* Eastern Cooperative Oncology Group performance status, *TRG* tumor regression grade, *pCR* pathologic complete remission, *PFS* progression-free survival, *OS* overall survival, *M* male, *F* female, *CR* complete remission, *PR* partial remission, *SD* stable disease, *PD* progressive disease

### Adverse events

Regarding hematological adverse events, 6 (37.5%), 5 (31.3%), 2 (12.5%), and 2 (12.5%) patients experienced white blood cell (WBC) reduction, neutrophil decreased, thrombocytopenia, and anemia (Table 3). Regarding non-hematologic adverse events, 6 (37.5%), 4 (25.0%), 3 (18.8%), 3 (18.8%), 2 (12.5%), 2 (12.5%), 1 (6.3%), and 1 (6.3%) patients suffered from reactive cutaneous capillary endothelial proliferation (RCCEP), nausea or vomiting, decreased appetite, baldness, diarrhea, constipation, fatigue, and rash.

**Table 3:** Adverse events during neoadjuvant therapy

Adverse events	ESCC patients (*N* = 16)
Hematologic, no. (%)	
WBC decreased	6 (37.5)
Neutrophil decreased	5 (31.3)
Anemia	2 (12.5)
Thrombocytopenia	2 (12.5)
Non-hematologic, no. (%)	
RCCEP	6 (37.5)
Nausea or vomiting	4 (25.0)
Decreased appetite	3 (18.8)
Baldness	3 (18.8)
Diarrhea	2 (12.5)
Constipation	2 (12.5)
Fatigue	1 (6.3)
Rash	1 (6.3)

*ESCC* esophageal squamous cell carcinoma, *WBC* white blood cell, *RCCEP* reactive cutaneous capillary endothelial proliferation

### Discussion

In present study, it was revealed that (1) after the neoadjuvant therapy, ORR was 81.3% and DCR was 100%. (2) In surgery, the pCR rate and R0 resection rate were 31.3 and 93.8%, respectively. (3) The 1-year PFS and OS were 83 and 90.9%, respectively. (4) All adverse events were slight and tolerable.

Currently, the chemotherapy and radiotherapy are recommended neoadjuvant therapy for locally advanced ESCC patients [6–8]. What’s more, some recent studies point out that targeted drugs (such as apatinib, pembrolizumab, and nivolumab) combined with chemotherapy could possibly exhibit an even better efficacy for the locally advanced ESCC patients. However, there is little evidence around neoadjuvant camrelizumab combined with chemotherapy in treating locally advanced ESCC patients. Subsequently, the current study turn attention to camrelizumab, a PD-1 immune checkpoint inhibitor, which blocks the binding site of PD-1 expressed on activated T lymphocytes, B cells, and natural killer cells to PD-L1 overexpressed on certain cancer cells. So far, several studies report that camrelizumab combined with chemotherapy has been used in adjuvant and second-line therapy of locally advanced ESCC and exhibits a promising antitumor efficacy [17, 18]. However, the clinical value of neoadjuvant camrelizumab combined with chemotherapy in locally advanced ESCC is still unclear. In present study, patients receiving neoadjuvant camrelizumab combined with chemotherapy reached an ORR of 81.3% and DCR of 100% in locally advanced ESCC patients. Possible explanations might be that (1) camrelizumab blocked the binding of PD-1 to PD-L1; promoted the activation of monocytes, T cells, B cells, dendritic cells, and tumor-infiltrating lymphocytes to exert tumor suppressive function; and furthermore contributed to better therapeutic response [23]. (2) Besides, chemotherapy might help to activate tumor-specific T cells by promoting tumor antigen presentation and by destroying immunosuppressive factors and further promotes the antitumor efficacy of camrelizumab [24]. Hence, synergic effect of cytotoxic chemotherapy plus camrelizumab probably results in a favorable therapeutic response.

Regarding pathological response, previous studies exhibited that an R0 resection rate was 96.3% and a pCR rate was 33.3% in patients with locally advanced ESCC receiving neoadjuvant nivolumab and pembrolizumab plus chemotherapy [9]. Likewise, in another study, pCR rate was 34.21%; meanwhile, R0 resection rate was 92.11% in locally advanced ESCC patients receiving neoadjuvant camrelizumab and pembrolizumab plus chemotherapy [25]. In this present study, 5 (31.3%), 6 (37.5%), 3 (18.8%), and 2 (12.5%) patients reached TRG 0, TRG 1, TRG 2, and TRG 3, respectively; 31.3% patients had pCR; meanwhile, 93.8% patients were witnessed with R0 resection. Possible explanations were as follows: (1) Camrelizumab may promote the cytotoxic effect of chemotherapy in tumor cells through elevating the chemosensitivity in patients with locally advanced ESCC; therefore, camrelizumab plus chemotherapy might present a better tumor suppressive effect than neoadjuvant chemotherapy alone, contribute to improve R0 resection rate, pCR rate, and tumor regression effect [26]. (2) Camrelizumab suppresses tumor angiogenesis and stimulates antineoplastic activities, which could be facilitated by chemotherapy [27]. Hence, the neoadjuvant camrelizumab plus chemotherapy might elevate the antitumor efficiency and further achieve a favorable R0 resection rate, pCR rate, and tumor regression effect in patients with locally advanced ESCC.

So far, few studies investigate the survival profile of neoadjuvant PD-1 inhibitors plus chemotherapy in patients with locally advanced ESCC. In the present study, it was revealed that with neoadjuvant camrelizumab plus chemotherapy treatment, the mean PFS and OS were 18.3 months and 19.2 months with a 1-year PFS of 83% and 1-year OS of 90.9%, respectively. These findings might be explained by the following hypotheses: (1) Camrelizumab may promote the cytotoxic effect of chemotherapy on tumor cells through increasing the chemosensitivity. Furthermore, camrelizumab combined with chemotherapy might present a better tumor suppressing efficacy particularly in less immunogenic, chemo-sensitive tumors [26]. (2) Neoadjuvant camrelizumab combined with chemotherapy achieves a good treatment response and satisfactory pCR rate, as discussed above, which may contribute to improve prognosis in present research.

Concerning camrelizumab for the treatment of gastric and esophagus cancer, the major adverse events were discovered in the skin, gastrointestinal tract, endocrine glands, liver, and lung; meanwhile, most of them were mild and manageable [28]. As to the current study, the major adverse events include white blood cell (WBC) reduction, neutrophil decreased, RCCEP, nausea, or vomiting, most of which were mild and controllable. It is indicated that neoadjuvant camrelizumab combined with chemotherapy was well-tolerant as the neoadjuvant therapy of locally advanced ESCC.

Limitations were as followed: (1) The relatively small sample size of eligible patients might potentially reduce the reliability of the results. (2) As this was a single-arm study, it lacks a control group; hence, further randomized, controlled study would be desirable. (3) Follow-up period was relatively short, and further study with a longer follow-up period was necessary to determine the long-term efficacy of neoadjuvant camrelizumab combined with chemotherapy in patients with locally advanced ESCC. (4) The economic evaluation of camrelizumab combined with chemotherapy was neglected in the current study, which needs further exploration. (5) In the present study, patients’ selection bias might exist to affect the survival profile.

## Conclusions

Collectively, neoadjuvant camrelizumab plus chemotherapy exhibits good efficacy and acceptable tolerance in treating patients with locally advanced ESCC, while further validation of the efficacy in larger cohort is needed.

## Data Availability

The datasets generated and/or analyzed during the current study are not publicly available because the data are confidential patient data but are available from the corresponding author upon reasonable request.

## Abbreviations

ESCC: Esophageal squamous cell carcinoma
TRG: Tumor regression grade
pCR: Pathological complete remission
PFS: Progression-free survival
OS: Overall survival
ORR: Objective remission rate
DCR: Disease control rate
PD-1: Programmed cell death protein 1
PD-L1: Programmed cell death ligand 1
CT: Computed tomography
CR: Complete remission
PR: Partial remission
SD: Stable disease
IQR: Interquartile range
